# Comment on: The Benefits of Resistance Training in Obese Adolescents: A Systematic Review and Meta-analysis

**DOI:** 10.1186/s40798-022-00543-7

**Published:** 2023-02-08

**Authors:** Fan Zhang

**Affiliations:** Department of Nephrology, Longhua Hospital Shanghai University of Traditional Chinese Medicine, Shanghai, China

## Dear Editor

The study by Ribeiro et al. [1] described the benefits of resistance training in obese adolescents, but it is necessary to highlight some methodological issues to facilitate subsequent studies.

First, as a matter of detail, I suggest that authors should consider using a combination of medical subject terms (Mesh) and keywords when developing their search strategy, such as adding “resistance training”[Mesh] (https://www.ncbi.nlm.nih.gov/mesh/?term=resistance+training) and “Adolescent”[Mesh] (https://www.ncbi.nlm.nih.gov/mesh/68000293) to the current search strategy. It is possible that some crucial studies were missed by using keywords alone.

**Fig. 1:**
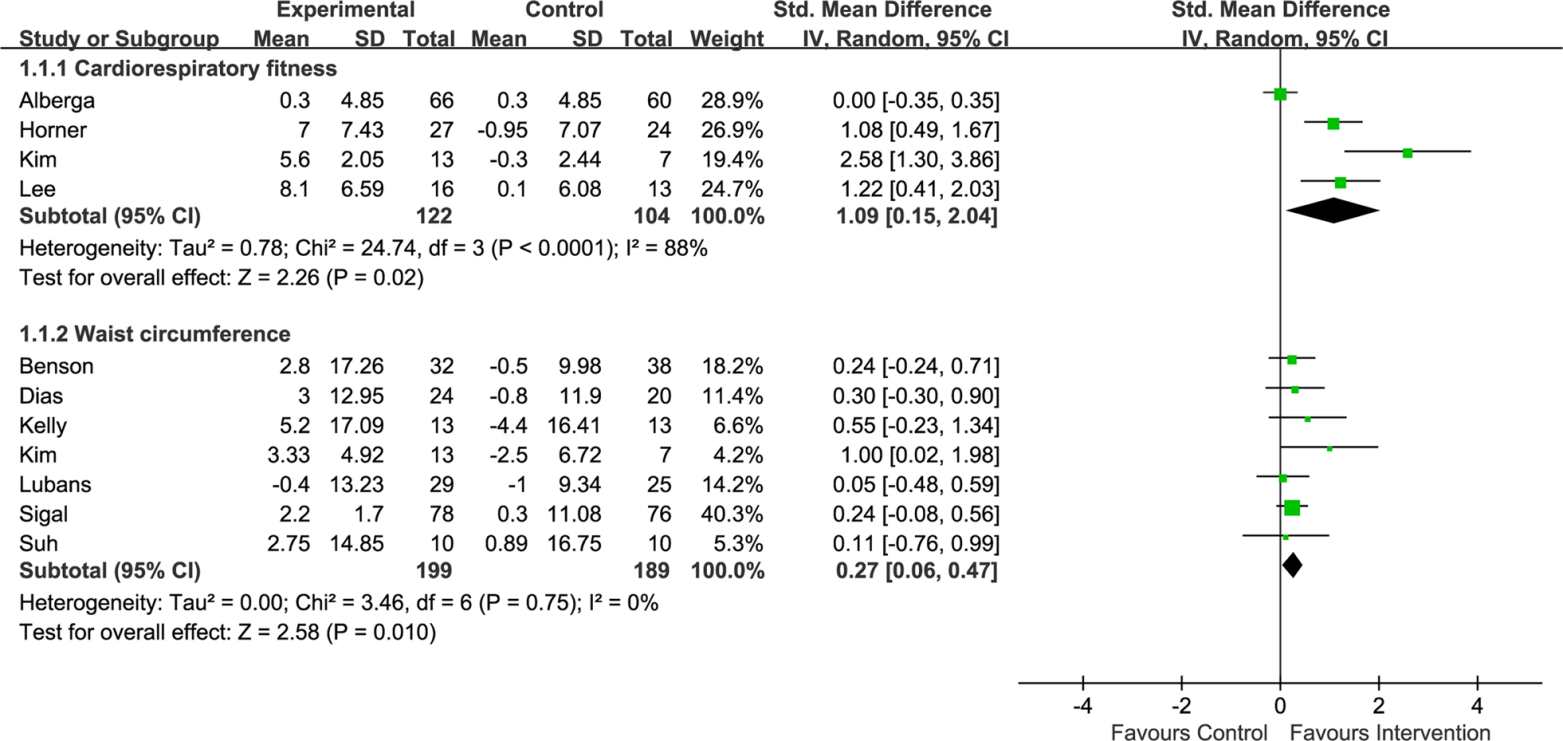
Forest plot of comparison for cardiorespiratory fitness and waist circumference

Second, there is an error in the forest plot drawn by the authors. Muscle strength, body mass index, cardiorespiratory fitness, waist circumference, lean mass, body fat, and insulin sensitivity are not the same type of outcome, but the authors combined the weights of all studies, reducing the degree of influence of each study on the overall outcome. The correct approach should be (in the case of cardiorespiratory fitness and waist circumference): tick “subtotals only” in “analysis details” of “properties” in the interface of Revman software forest plot” so that each result is independent, as shown in Fig. 1. Although the pooled effect size remains the same, the weight is increased.

Third, the study process for this systematic review and meta-analysis was incomplete. The study included > 10 trials, and publication bias should be determined by plotting funnel plots [2] and using sensitivity analysis to determine the stability of the results. Based on these results, it is also recommended to use the Grading of Recommendations Assessment, Development, and Evaluation approach [3] to assess the evidence for resistance training on different outcomes to facilitate clinical practice recommendations.

## Data Availability

Not applicable.
